# Supplementary material for: Complexome profiling of the Chlamydomonas psb28 mutant reveals TEF5 as an early PSII assembly factor
Source: Plant Cell. 2025 Mar 17;37(6):koaf055. doi: 10.1093/plcell/koaf055 (PMC12164590; doi:10.1093/plcell/koaf055)
Supplement: koaf055_Supplementary_Data [file koaf055_supplementary_data.zip › psb28_interactive.zip!/plots/intensity/qualityPlots/peptides/RAB11_Cre03.g189250.t1.2.html]

### Cre03.g189250.t1.2

Trivial name: RAB11  
SeqCoverage [median]: 69.90  
Mass: 24.18 kDa  
  
 

| Sequence | MissedCleavage | Mass [Da] | wtIntensity | muIntensity | Score |
| --- | --- | --- | --- | --- | --- |
| AITSAYYR | 0 | 943.48 | 1341.44 | 6355.61 | 114.78 |
| AQIWDTAGQER | 0 | 1273.61 | 3784.99 | 1469.85 | 152.97 |
| DHADSNIVIMLVGNK | 0 | 1624.82 | 375.25 | 6196.53 | 84.57 |
| DVIVIDNAHDDGGK | 0 | 1466.70 | 0.00 | 762.91 | 62.29 |
| DVIVIDNAHDDGGKK | 1 | 1594.80 | 0.00 | 1721.83 | 47.53 |
| EGLSFIETSALESTNVEK | 0 | 1952.96 | 420.20 | 0.00 | 59.90 |
| FTRNEFSLESK | 1 | 1356.67 | 1012.19 | 4995.22 | 86.46 |
| GAVGALLVYDITK | 0 | 1318.75 | 3644.16 | 5160.68 | 96.02 |
| NEFSLESK | 0 | 952.45 | 823.28 | 4116.30 | 96.49 |
| SHKPDDDYDYLFK | 1 | 1641.73 | 2501.20 | 21312.42 | 155.98 |
| SIQVDGK | 0 | 745.40 | 4025.61 | 12255.28 | 99.00 |
| STIGVEFATR | 0 | 1079.56 | 2224.73 | 9599.48 | 113.71 |
| SVTFENVER | 0 | 1079.52 | 3270.02 | 9848.01 | 113.71 |
| VLDSEDNRPK | 1 | 1171.58 | 3667.20 | 14197.81 | 98.05 |
| VVLIGDSGVGK | 0 | 1042.60 | 15371.32 | 34589.60 | 108.98 |
|  |  |  | 42461.6 | 132581.5 |  |
